# Carbon Double Coated Fe_3_O_4_@C@C Nanoparticles: Morphology Features, Magnetic Properties, Dye Adsorption

**DOI:** 10.3390/nano12030376

**Published:** 2022-01-24

**Authors:** Chun-Rong Lin, Oxana S. Ivanova, Irina S. Edelman, Yuriy V. Knyazev, Sergey M. Zharkov, Dmitry A. Petrov, Alexey E. Sokolov, Eugeniy S. Svetlitsky, Dmitry A. Velikanov, Leonid A. Solovyov, Ying-Zhen Chen, Yaw-Teng Tseng

**Affiliations:** 1 Department of Applied Physics, National Pingtung University, Pingtung City 90003, Taiwan; stp92065@gmail.com (Y.-Z.C.); chargedw@yahoo.com.tw (Y.-T.T.); 2 Kirensky Institute of Physics, FRC KSC SB RAS, 660036 Krasnoyarsk, Russia; ise@iph.krasn.ru (I.S.E.); yuk@iph.krasn.ru (Y.V.K.); zharkov@iph.krasn.ru (S.M.Z.); irbiz@iph.krasn.ru (D.A.P.); alexeys@iph.krasn.ru (A.E.S.); evgenij.svetlitsky@yandex.ru (E.S.S.); dpona1@gmail.com (D.A.V.); 3 Institute of Engineering Physics and Radioelectronics, Siberian Federal University, 660041 Krasnoyarsk, Russia; 4 Institute of Chemistry and Chemical Technology, FRC KSC SB RAS, 660036 Krasnoyarsk, Russia; leosol@icct.ru

**Keywords:** core-shell Fe_3_O_4_@C nanoparticles, core-shell Fe_3_O_4_@C@C nanoparticles, magnetic properties, dyes adsorption

## Abstract

This work is devoted to the study of magnetic Fe_3_O_4_ nanoparticles doubly coated with carbon. First, Fe_3_O_4_@C nanoparticles were synthesized by thermal decomposition. Then these synthesized nanoparticles, 20–30 nm in size were processed in a solution of glucose at 200 °C during 12 h. The morphology and features of the magnetic properties of the obtained hybrid nanoparticles were characterized by transmission electron microscopy, differential thermo-gravimetric analysis, vibrating sample magnetometer, magnetic circular dichroism and Mössbauer spectroscopy. It was shown that the magnetic core of Fe_3_O_4_@C nanoparticles was nano-crystalline, corresponding to the Fe_3_O_4_ phase. The Fe_3_O_4_@C@C nanoparticles presumably contain Fe_3_O_4_ phase (80%) with admixture of maghemite (20%), the thickness of the carbon shell in the first case was of about 2–4 nm. The formation of very large nanoparticle conglomerates with a linear size up to 300 nm and of the same regular shape is a remarkable peculiarity of the Fe_3_O_4_@C@C nanoparticles. Adsorption of organic dyes from water by the studied nanoparticles was also studied. The best candidates for the removal of dyes were Fe_3_O_4_@C@C nanoparticles. The kinetic data showed that the adsorption processes were associated with the pseudo-second order mechanism for cationic dye methylene blue (MB) and anionic dye Congo red (CR). The equilibrium data were more consistent with the Langmuir isotherm and were perfectly described by the Langmuir–Freundlich model.

## 1. Introduction

Magnetic nanoparticles (NPs) remain a hot trend in condensed matter physics despite the relatively long history of their research, primarily due to the many new questions that they pose for fundamental science and their applications in various modern technologies. A large number of synthesis methods leads to a large variety of NPs properties. As a rule, NPs tend to agglomerate in order to reduce their surface energy and modification of the NPs surface is a solution to prevent this phenomenon. In general, surface modification can be accomplished by physical and/or chemical adsorption of the desired molecules to coat the surface. Carbon shell is one of the universal coatings for NPs, which is used in many cases. It provides exceptional chemical stability and protects the magnetic core from oxidation, allowing further functionalization of the carbon surface. Core-shell nanostructures consisting of the iron oxide magnetic core coated with non-magnetic carbon demonstrate unique properties and technological capabilities [1,2,3,4,5,6].

Sorption of different water pollutants is one of the widespread fields of magnetic NPs application. An important advantage of using magnetic NPs in this field is a possibility to extract them easily from the medium by applying a magnetic field. Among others, Fe_3_O_4_@C NPs are the most attractive candidates for this purpose, since they combine good sorption properties of carbon with high magnetic properties of magnetite (Fe_3_O_4_). Many authors have devoted their efforts to the application of Fe_3_O_4_@C NPs as adsorbents of various substances. These NPs were used as sorbents of heavy metals (Cu, Ni, Co, and Cd) [7], and the carbon-coated surface of Fe_3_O_4_ was functionalized with polyacrylamide to increase the removal efficiency. In [8], it was shown that the carbon layer was responsible for the effective adsorption of the polycyclic aromatic hydrocarbons by Fe_3_O_4_@C NPs. In [9], Fe_3_O_4_@C NPs obtained by the light hydrothermal reaction of glucose with iron were used as the magnetic solid-phase extraction sorbent of brominated flame retardants and pentachlorophenol. A number of publications were devoted to the study of dye adsorption by Fe_3_O_4_@C NPs. Kinetics, equilibrium and thermodynamics of dye adsorption by the one step fabricated Fe_3_O_4_@C NPs were studied in [10]. Much research was conducted on the removal of methylene blue and other dyes from water [11,12,13,14,15,16]. The high adsorption capacity of Fe_3_O_4_@C nanoparticles together with easy magnetic separation and the possibility of reuse noted in these works stimulate the search for new technological solutions for further improving the characteristics of adsorbents based on these kinds of NPs.

Several authors considered Fe_3_O_4_@C nanostructures as the basis for anode materials of lithium-ion batteries [2,3,4]. The hierarchical Fe_3_O_4_@C microspheres obtained by the self-assembling of microalgae were shown in [2] to be excellent as anode materials for lithium-ion batteries. Fe_3_O_4_ nanoparticles mechanically mixed with carbon fibers were used in a solid-state battery with a sulfide electrolyte; they were characterized by high capacitance along with very good recovery [3]. Recently, the authors of Ref. [4] have prepared, possibly for the first time, the double carbon coated Fe_3_O_4_@C@C NPs using the solvothermal method with the following heat treatment. They show that the multilayer carbon coating essentially improved the discharge capacity and cycling stability of NPs. Such an approach seems to be very promising, since the double carbon coating can improve the performance of nanostructures or even give them new properties that are useful for a variety of applications. Having this in mind, we fabricated Fe_3_O_4_ NPs doubly coated with carbon using other simpler and cheaper method compared to that used in [4]. Once coated, Fe_3_O_4_@C NPs of 20–30 nm in size synthesized with the thermal decomposition method were processed in a solution of glucose which led to an unexpected phenomenon—the NPs self-assembled into large conglomerates of the regular shape (they looked like pillows) and of about 300 nm in size. The detailed study of morphology, structure, and magnetic properties of the obtained NPs, both once- and double-coated with carbon, and the estimation of their application perspectives were the aims of the present investigation. The morphology and structure of the obtained samples were studied with XRD and transmission electron microscopy. The magnetic properties were investigated with a vibrating sample magnetometer, Mössbauer spectroscopy, and magnetic circular dichroism (MCD) in the visible and near IR spectral ranges. We also considered the possibility of using the obtained nanostructures as adsorbents of organic dyes from water. From the analysis of the current literature (to be presented further), it became clear that the efforts of researchers have so far been concentrated, mainly, on the study of the adsorption of a cationic dye—methylene blue (MB). Anionic dyes have received much less attention; in particular, we failed to find any work on the adsorption of an anionic Congo red (CR) on carbon-based structures, although CR is a reference dye—analogous to endotoxins (bacterial toxic substances). So, here we carried out a comparative study of the MB and CR adsorption from water solutions on the synthesized Fe_3_O_4_@C and Fe_3_O_4_@C@C samples.

## 2. Materials and Methods

### 2.1. Synthesis Procedure

Iron (III) nitrate nonahydrate, Fe(NO_3_)_3_·9H_2_O and oleic acid (C_18_H_34_O_2_) were obtained from Sigma-Aldrich, ethanol (CH_3_CH_2_OH) (>95%) and hexane (>95%) were obtained from Fullin Nihon Shiyaku Bio-chemical Ltd. (Taiwan, China), oleylamine (C_18_H_35_NH_2_) was purchased from Acros Organics (Geel, Belgium), glucose (C_6_H_12_O_6_) was purchased from AENCORE (Box Hill, Australia). All the chemicals were used without further purification.

Two stages were used to synthesize nanocomposites. In the first stage, the initial Fe_3_O_4_@C nanoparticles were synthesized by a one-step process described earlier in Ref. [17]. In a typical process, 8 mmol of Fe(NO_3_)_3_·9H_2_O, 20 mL of oleic acid, and 5 mL of oleylamine were mixed in a three-neck flask. To remove water, the mixture was heated to 140 °C and kept at this temperature for 60 min, then the temperature was raised to 240 °C and maintained for 1 h. The final temperature of the reaction was 380 °C for 1 h. This temperature was chosen because it was shown to provide formation of a single-phase magnetic core of particles corresponding to magnetite Fe_3_O_4_ in [17]. The reactions were carried out under an argon flow, and the temperature was controlled by a thermocouple. After cooling the mixture to room temperature, nanoparticles were separated from the suspension with a magnetic field. To completely remove excess organic solvent and by-products from the samples, they were separated by magnetic decantation and washed several times with hexane.

**Figure 1:**
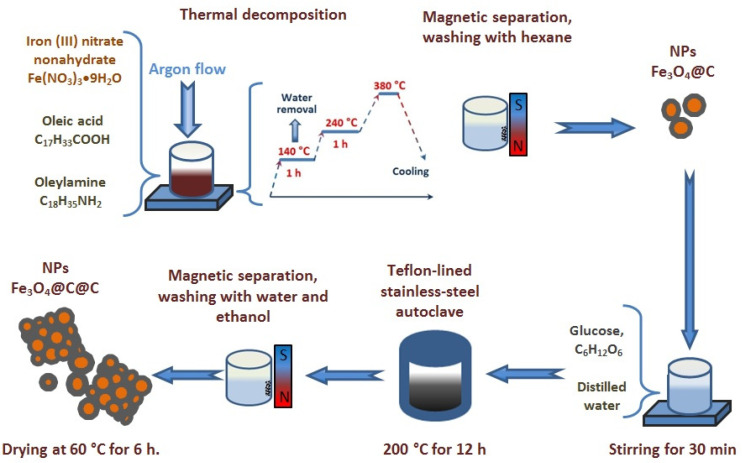
Schematic illustration of the NPs preparation steps. The equipment used is shown in a simplified manner.

In the second stage, 1.0 g of glucose was dissolved in 30 mL of distilled water under stirring for 15 min., followed by the addition of 0.2 g of Fe_3_O_4_@C NPs synthesized at the first stage. After stirring for 30 min, the final mixture was transferred to a Teflon-lined stainless-steel autoclave (50 mL capacity) and heated at 200 °C for 12 h, and then allowed to cool to room temperature. The black products were separated by a magnet and were washed several times with water and ethanol. Finally, the solid products were dried at 60 °C for 6 h. The schematic illustration of the NPs preparation steps is presented in Figure 1.

### 2.2. Characteristic Methods

The powder X-ray diffraction (XRD) data were obtained on a PANalytical X’Pert PRO diffractometer equipped with a solid state detector PIXcel and a graphite monochromator using Cu Kα radiation over the 2θ range 10–135°.

The morphology and elemental composition of NPs were examined using a JEM-2100 transmission electron microscope (JEOL Ltd., Tokyo, Japan) operating at an accelerating voltage of 200 kV equipped with an Oxford Instruments energy dispersive spectrometer (EDS). Selected-area electron diffraction (SAED) was used to determine the structure of NPs.

The differential Scanning Calorimeter DSC 204 F-1 Phoenix (NETZSCH, Selb, Germany) was used to remove the possible admixture of the excess organic solvent and by-products from the NP surfaces.

The Mössbauer effect spectra of the samples were obtained on an MS-1104Em spectrometer in transmission geometry with a ^57^Co (Rh) radiation source at 300 K. The spectra processing was performed by fitting the model spectrum to the experimental one by varying the entire set of hyperfine parameters by the least squares method in the linear approximation.

The NPs magnetization dependences on the external magnetic field were studied with the laboratory made vibrating sample magnetometer [18,19].

For the MCD measurements in transmitted light, transparent composite plates consisting of the dielectric silicone-based adhesive (“Ray her” art. Nr. 3,338,100 80 mL) mixed with NPs in a weight ratio of 100/0.3 were prepared. The low magnetic NPs concentration allowed us to avoid interaction between them. MCD was measured with the laboratory made device in normal geometry: directions of the magnetic field vector and the light wave propagation were normal to the plane of the plates. The modulation of the light wave circular polarization state from the right to the left relative to the direction of the magnetic field was used. MCD was measured as the difference between the optical densities of the sample for the right (D_+_) and left (D_−_) polarized waves: ∆D/D = (D_+_−D_−_)/(D_+_+D_−_) in the spectral range 1.25–3.5 eV in a magnetic field up to 1.3 T at temperatures 300 and 100 K. The measurement accuracy was about 10^–4^, and the spectral resolution was 20–50 cm^−1^, depending on the wavelength.

Changes in the absorption spectra of the dye solutions by NPs were recorded with the UV/VIS circular dichroism spectrometer SKD-2MUF (OEP ISAN, Moscow, Russia). Quartz cells with an optical path length of 5 mm were used.

## 3. Results

### 3.1. NPs Structure and Morphology

**Figure 2:**
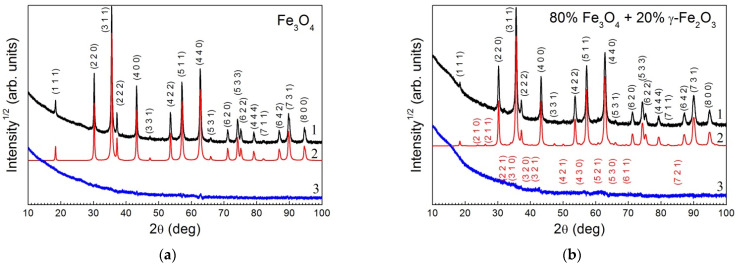
Observed (1), calculated (2), and different (3) XRD profiles after DDM refinement for the initial Fe_3_O_4_@C sample (**a**) and for the double carbon coated Fe_3_O_4_@C@C sample (**b**). The crystal planes corresponding to the observed reflections are marked in black for magnetite (**a**) and red for maghemite (**b**). Most of the reflexes coincide for both compounds (**a**,**b**), but some of them of very low intensity are characteristic only of maghemite (**b**).

The powder X-ray diffraction (XRD) patterns are shown by curves 1 in Figure 2a,b for the initial Fe_3_O_4_@C NPs and for the double carbon coated Fe_3_O_4_@C@C NPs, correspondingly.

The quantitative phase analysis and full-profile refinement of crystal lattice parameters were made using the Rietveld method [20] with the derivative difference minimization (DDM) technique [21]. The calculated XRD patterns and differences between the observed and calculated ones are presented in Figure 2 by curves 2 and 3, correspondingly. The initial sample consisted of pure magnetite, Fe_3_O_4_, with lattice parameter 0.83946(1) nm (PDF 4+ card #04-005-4319, Fd-3m, a = 0.8396 nm) and estimated average crystallite size 43 nm. The phase composition of Fe_3_O_4_@C@C sample included 80% magnetite (Fe_3_O_4_) and 20% maghemite (γ-Fe_2_O_3_) with the cubic lattice parameters 0.8396(1) nm for Fe_3_O_4_ (PDF 4+ card #04-005-4319, SG: Fd-3m) and a = 0.8352(1) nm for Fe_2_O_3_ (PDF 4+ card #00-039-1346, SG: P4132). The estimation of the crystallite sizes in this case is difficult due to the presence of two phases with very close lattice parameters. However, judging by the width of the reflexes, it can be stated that no noticeable change in this value is observed. It would be possible to assume the formation of iron carbide at the boundaries of iron and carbon during the synthesis of samples. However, the XRD patterns contain only reflexes characteristic of iron oxides with a cubic structure.

**Figure 3:**
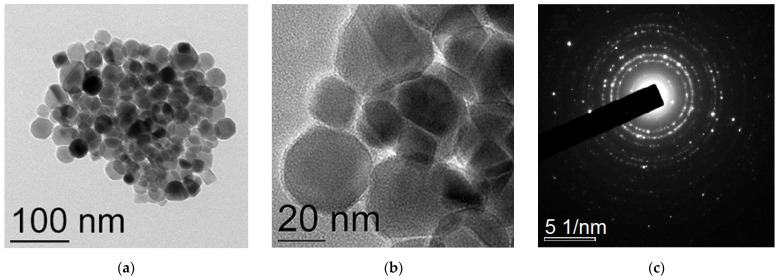
TEM images (**a**,**b**) with different magnification and SAED pattern (**c**) of the Fe_3_O_4_@C one step synthesized NPs.

**Figure 4:**
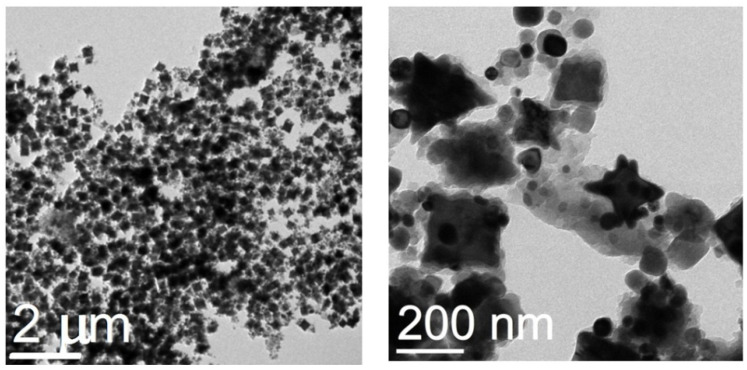
TEM images of the two steps synthesized Fe_3_O_4_@C@C NPs with different magnification.

TEM images of the initial Fe_3_O_4_@C NPs prepared in the one-stage process are presented in Figure 3a,b. Most of the nanoparticles have a spherical shape; faceted and polygonal shaped NPs are observed too. The NPs size varies between 20 and 40 nm, which corresponds to the average crystallite size determined with the XRD analysis. Thin carbon amorphous shells around each NP are seen well in the TEM image (Figure 3b). The electron diffraction patterns (Figure 3c) correspond to the Fd-3m phase characteristic of Fe_3_O_4_ also in accordance with the XRD data. After treatment in the glucose solution, there appear many very large particles up to 500 nm in size, of a polyhedron shape (Figure 4). They can both be conglomerates of smaller particles and homogeneous ones. The fact that the particle sizes are many times larger than the average crystallite size testifies to the first assumption. In any case, these formations are definitely not single crystals. Separate spherical or rectangular NPs with sizes similar to those in the initial sample are observed. These NPs cluster near large particles. The picture looks like they do not have enough time to unite into large particles.

To remove excess organic solvent and by-products from the surface of NPs, we carried out thermal treatment of the NPs powder in the course of thermo-gravimetric analysis (TG) combined with differential scanning calorimetry (DSC) when the sample was heated up to 600 °C in the argon atmosphere.

**Figure 5:**
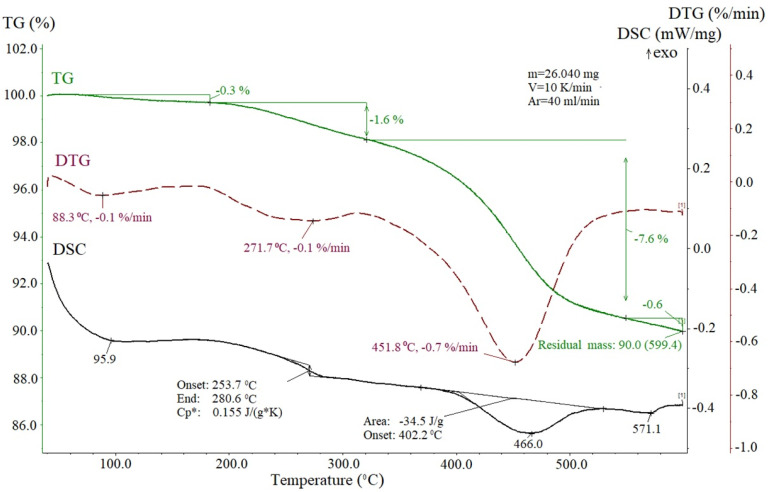
Thermogravimetric (TG) and differential thermogravimetric (DTG) as well differential scanning calorimetric (DSC) profiles of the Fe_3_O_4_@C@C NPs.

As seen in Figure 5, the process of thermal destruction took place in, at least, two stages. The first stage (temperature region 200–300 °C) was probably associated with the destruction of the outer carbon layer, leading to a small decrease of 1.6% of the original mass (TG curve) and to a change in specific heat (DSC curve) due to the partial carbon withdrawal. The second stage (300–500 °C) of the process proceeds more intensively—the weight loss is 7.6%, and the weight loss rate is 0.7%/min, and is accompanied by an endothermic effect with a maximum at 466 °C, with a change in enthalpy of 34.5 J/g.

**Figure 6:**
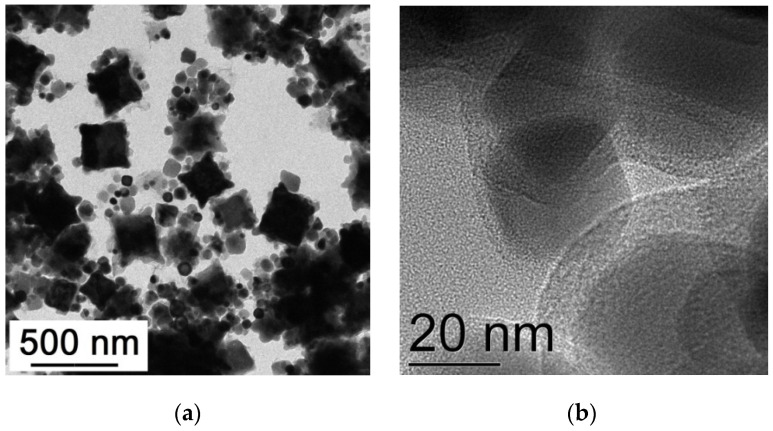
TEM (**a**) and HRTEM (**b**) images of the Fe_3_O_4_@C@C NPs after annealing.

Such a small total mass loss is typical for similar nanostructures and could indicate the thinness of the carbon layer on the magnetic core. However, the carbon layers around the magnetite core are rather thick in our case as is seen from TEM image of the sample fragments subjected to the thermal treatment (Figure 6b). One can assume that the small mass loss is due to the removal of carbon only from the surface areas of large conglomerates.

**Figure 7:**
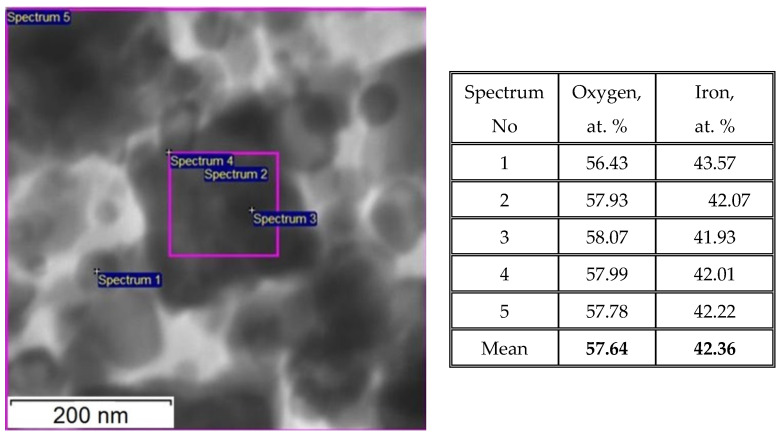
STEM image and Table with the EDS data on the oxygen and iron concentrations in different points (2, 3) inside the pink square, in small spherical particle (1) and average over the pink square (4) and over the whole field of the Fe_3_O_4_@C@C NPs.

The sample Fe_3_O_4_@C@C morphology did not change after thermal treatment (compare Figure 4 and Figure 6a); it becomes clearer that the polyhedrons acquire an almost ideal shape and blurry foggy areas around small particles disappear. The latter can be associated with glucose residues that disappear when heated. Thus, the formation of large stable polyhedrons of the same shape and similar size (200–300 nm) is the main feature of the synthesized product. The question arises about the polyhedrons elemental composition. As follows from the EDS data analysis (see Figure 7) iron is distributed approximately evenly over the entire area of the polyhedron (see points 2, 3, 4 in square inside large particle). The iron concentration in these points is close to that in the stoichiometric Fe_3_O_4_ (Fe—42.86 at. %, O—57.14 at. %).

Note that the formation of Fe_3_O_4_ nanoaggregates is highly desirable for applications as electrode materials in lithium batteries [22], and several authors have undertaken efforts to synthesize this kind of NP using different templates. For example, porous Fe_3_O_4_/C nanoaggregates were fabricated by the multi-step template hydrothermal method in [22]. Carbon polyhedrons derived from a metal organic framework consisting of zeolite imidazolate and Co^2+^ metal centers were used as templates. The nanoaggregates obtained by this method looked like irregular polyhedrons (somewhat similar to that shown in Figure 4) up to 500 nm in size, consisting of many small particles. On the other hand, the authors of Ref. [23] synthesized Fe_3_O_4_ NPs coated with glucose using the one-step hydrothermal method with one precursor FeCl_3_ in sucrose. They controlled the NPs size from 4 to 16 nm by changing the feeding concentration of sucrose. However, no NPs agglomeration was observed.

In our case, there was no specially prepared template. It can be assumed that under the technological conditions used—heating at a constant not very high temperature over a rather long period of time—glucose forms structures serving as a kind of framework. Glucose decomposition at heating was shown in [24] to start at 188 °C and reach maximum rate at 220 °C (Table 1 in Ref. [24]). Moreover, this process can make some contribution to the curves shown in Figure 5. Constant heating at 200 °C for 12 h leads to the gradual decomposition of glucose with the formation of free carbon and, in addition, stimulates the association of the glucose molecules into conglomerates in places of random increase in its concentration in solution. At the same time, the initial Fe_3_O_4_@C NPs can contain the residual molecules of oleylamine and oleic aside in their surfaces and, as a consequence, be accumulated around these conglomerates. These considerations make it possible to understand the reason for the identical shape of the NPs aggregates shown in Figure 4 and Figure 6.

### 3.2. Mössbauer Spectra

**Figure 8:**
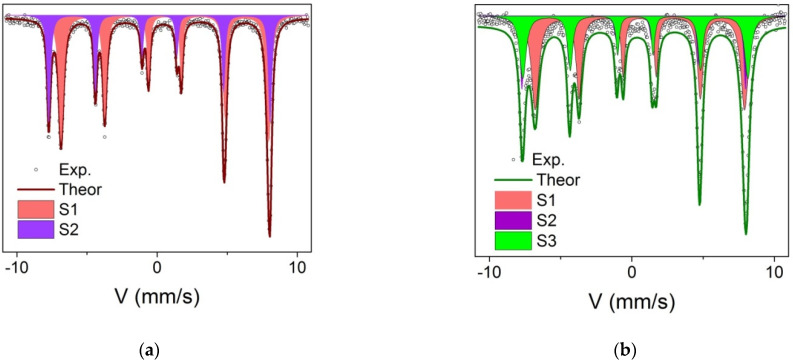
Mössbauer spectra of Fe_3_O_4_@C (**a**) and Fe_3_O_4_@C@C (**b**) samples measured at 300 K.

The room temperature Mössbauer spectra of the samples Fe_3_O_4_@C and Fe_3_O_4_@C@C presented in Figure 8 testify to the NPs ferromagnetic or blocked superparamagnetic (SP) state in both cases: the spectra are well approximated by two, in the first case, and three, in the second case, sextets (Table 1). Doublets characteristic of the SP state are absent at all. The isomer shift (IS) values indicate iron Fe^2.5+^ in octahedral coordination. Such a charge state arises due to the fast electronic exchange between Fe^2+^ and Fe^3+^ cations in octahedral positions. This behavior is characteristic of magnetite above the Verwey temperature. The chemical shifts and hyperfine fields correspond to the parameters for magnetite. At the same time, the presence of maghemite, the γ-Fe_2_O_3_, phase is observed in the Fe_3_O_4_@C@C sample, which may be the result of oxidation magnetite particles during the second step of the NPs coating by carbon. The γ-Fe_2_O_3_ phase amount in sample Fe_3_O_4_@C@C corresponds to that determined with the XRD patterns analysis. It can be assumed that, similarly to [25], maghemite is formed on the surface of pure magnetite, forming a kind of shell.

### 3.3. NPs Magnetic Properties

**Figure 9:**
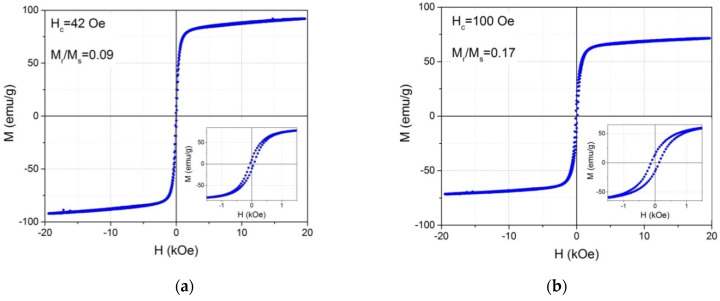
Magnetization loops of the samples Fe_3_O_4_@C (**a**) and Fe_3_O_4_@C@C (**b**) measured at room temperature. Insets: the same in lower magnetic field. M_s_ = 92.8 emu/g (**a**), 72.3 emu/g (**b**).

The magnetization curves are shown in Figure 9. They look like the superposition of the Langevin’s curves characteristic of SP nanoparticles and the hysteresis loops due to the blocked at room temperature superparamagnetic or ferromagnetic NPs. The difference between these and Mössbauer data is associated with the different times of the measurement methods. As is known, the relaxation time of the NPs magnetic moment τ is determined by the Neel–Brown equation [26].

(1) $$\tau =\tau_{0}exp\left(\frac{K_{eff}V}{k_{B}T}\right),$$

where *τ_0_*~10^−9^, *K_eff_* is the effective anisotropy constant, *V* the NPs volume, *k_B_* is the Boltzmann constant, *T* is the temperature. If the magnetic moments of the particles change their directions faster than the measurement time in a particular experiment, then in this experiment the superparamagnetic state of particles will be recorded, if slower, the blocked one. The characteristic measurement time with a magnetometer is about 1–100 s, while when recording the spectrum of the Mössbauer effect, each act of the γ-quant interaction with the matter is about 10^−8^ s. Thus, in the magneto-static experiment, a part of the NPs behaves as superparamagnetic and a part as blocked. Judging by the remnant magnetization M_r_/M_s_ and coercivity H_c_ (Figure 9), the quantity of blocked NPs is larger in the double carbon coated NPs. This means an increase of the blocking temperature of part of NPs in this sample, which can be due to an increase of the NPs volume or the effective anisotropy value. The last one can be associated with the appearance of maghemite shell around the magnetite core. Similarity to the magnetization curves of the initial Fe_3_O_4_@C sample (panel a in Figure 9) and to the sample with the double carbon coated NPs (panel b in Figure 9) testifies in favor of the fact that large polyhedra are not homogeneous crystals but are conglomerates of small particles in accordance with the XRD data.

The NPs magnetization is rather large in both cases, which is of importance for their application. It should be borne in mind that when determining the specific magnetization, the total mass of NPs was taken into account, since it was impossible to correctly separate the mass of the magnetic component and carbon. The saturation magnetization M_s_ was determined as the magnetization value in maximal magnetic field H = 20 kOe. For the initial sample Fe_3_O_4_@C, M_s_ = 92.8 emu/g which is equal to M_s_ of bulk Fe_3_O_4_ samples, 84 emu/g at room temperature [14], and essential greater than values presented by other authors for Fe_3_O_4_ NPs, for example, [19,21]. At the same time, the magnetization increase in the magnetite NPs coated with carbon comparing to uncoated NPs was noted by some authors [27,28]. To explain this enhancement, the authors of Refs. [27,28] proposed an ionic hypothesis of a polarized charge transfer to the A-site in the Fe_3_O_4_ NPs leading to a charge reduction at the A-site (i.e., Fe_A_^3+^ → Fe_A_^2+^). Since the resulting magnetization of the sample in Fe_3_O_4_ is due to the difference in the magnetic moments of Fe ions occupying octahedral and tetrahedral positions, the redistribution of Fe ions between oppositely magnetized sublattices in magnetite NPs is caused, for example, by technological conditions and can be one of the reasons for the different values of M_s_.

The magnetization of the double carbon coated NPs decreases noticeably, to 72.3 emu/g, which can be caused by the increase of the carbon amount in the sample and by the transformation of a part of magnetite into maghemite possessing lower magnetization value, M_s_ = 74 emu/g. Nevertheless, in this sample, the magnetization is quite high.

### 3.4. Magnetic Circular Dichroism

**Figure 10:**
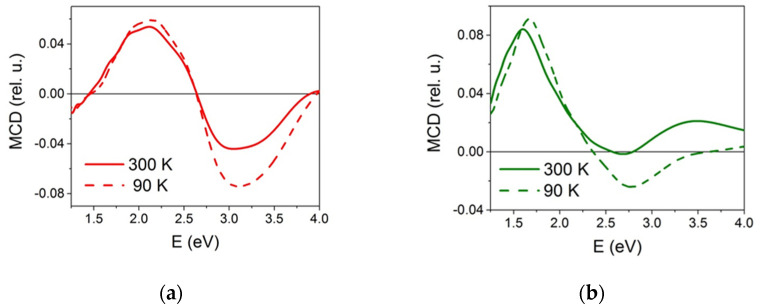
MCD spectra of Fe_3_O_4_@C (**a**) and Fe_3_O_4_@C@C (**b**) samples at 90 and 300 K.

The magnetic circular dichroism (MCD) spectra are presented in Figure 10 for two temperatures. The MCD spectrum of the initial sample Fe_3_O_4_@C coincides in shape with spectra of the imaginary part of the dielectric tensor off-diagonal ε_xy_^”^ component for bulk magnetite [29,30,31] and with the MCD spectrum of the magnetite thin films [32,33]. At that, the extrema in the spectrum can shift somewhat along the energy scale. Thus, in [33], when studying Fe_3_O_4_ NPs, a shift of the entire spectrum to the low-energy region was observed (the position of the maxima was at 1.7 and 2.6 eV, and the transition through zero was 2.35 eV).

For the Fe_3_O_4_@C@C sample, a similar shift is also observed, and if we take into account the presence of a certain amount of maghemite in the sample, mentioned above, the decrease in the magnitude of the negative maximum can presumably be explained by a contribution from maghemite, the spectrum of which is characterized by a positive maximum in the range 2.2–2.8 eV.

### 3.5. Application of Synthesized NPs for Dye Adsorption

**Figure 11:**
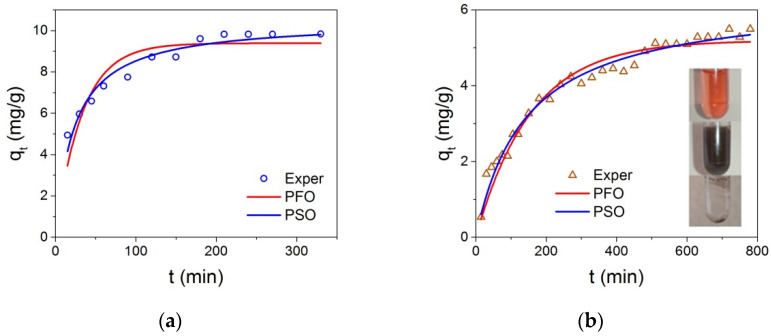
The *q_t_* dependence on the Fe_3_O_4_@C@C NPs contact time with MB (**a**) and CR (**b**) dyes, and results of the pseudo-first order (red lines) and pseudo-second order (blue lines) nonlinear modeling. The experimental conditions: *C_0_* = 30 mg/L, m(NPs) = 3 mg, *V* = 1.5 mL. Inset in panel b: the photographs of the initial CR water solution (at the top), the same solution with dispersed NPs (at the middle), and after gathering of NPs with the permanent magnet during a few tens of seconds (down).

Anionic–Congo red (CR) and cationic–methylene blue (MB) dyes dissolved in distilled water (pH = 5.5, T = 25 °C) were used to find out the adsorption capacity of the studied NPs. The solution temperature was controlled with the thermal coupled. The dye concentration in the water at each measurement stage was determined by the optical absorption of the solution at the wavelengths corresponding to the maxima in the optical absorption at 505 nm for CR, and 664 nm for MB. For the experiment, 3 mg of NPs was dispersed in 1.5 mL of an aqueous dye solution (MB or CR) and processed in an ultrasonic bath for 10 min. This treatment guaranteed a uniform distribution of particles throughout the entire volume of the solution. At the used power of the ultrasonic bath, the solution was heated from the initial temperature of 23 to 27 °C, so that the time average temperature was 25 °C. Then, magnetic NPs with the adsorbed dye were separated from the solution by applying a magnetic field and the optical absorption of the remaining solution was measured. Next, this solution was mixed again with the magnetic particles and the described procedure was repeated multiple times to obtain kinetic curves. We repeated the measurements three times and built graphs for the averaged values. The value of the NPs adsorption capacity at any given time *q_t_* (mg/g) was calculated as follows:

(2) $$q_{t}=\frac{\left(C_{0}-C_{t}\right)V}{m},$$

where *C*_0_ is the initial dye concentration in the solution and *C_t_* is the concentration of the dye at any time, V is the volume of the solution; and m represents the weight of the adsorbing NPs introduced into solution. The *q_t_*(t) dependencies are shown in Figure 11 for the MB and CR adsorption by Fe_3_O_4_@C@C NPs. In the case of Fe_3_O_4_@C NPs, the *q_t_*(t) curve shape is similar, *q_t_* magnitude is approximately half the size. In the case of MB, equilibrium adsorption capacity values *q_e_* under the conditions indicated in the captions to Figure 11 were: 5.6 and 10.0 mg/g for samples Fe_3_O_4_@C and Fe_3_O_4_@C@C, correspondingly. The adsorption rates were also different—the higher rate was observed for Fe_3_O_4_@C@C. Therefore, only results for the Fe_3_O_4_@C@C NPs will be presented below. It is seen that the ability of these NPs to adsorb the cationic dye is two times higher than that of the anionic dye at the initial dye concentration C_0_ = 30 mg/L.

Two main models are considered in current literature to describe kinetics of dye adsorption by NPs—the pseudo-first which is described as follows

(3) $$q_{t}=q_{e}\left(1-e^{-k_{1}t}\right)\;$$

and pseudo-second order

(4) $$q_{t}=\frac{q_{e}^{2}k_{2}t}{1+q_{e}k_{2}t},$$

where *k*_1_, *k*_2_ are the rate constants of the sorption reaction in the pseudo-first and pseudo-second orders, respectively. Nonlinear modeling was carried out using the OriginPro 2016 program (OriginLab Corporation, Northampton, MA, USA) utilizing the Levenberg–Marquardt iteration algorithm. The advantages of using this approach are discussed in [34]. The results of nonlinear modeling of the kinetic data are shown in Figure 11. Additionally, the correlation coefficients (*R*^2^) and the rate parameters obtained by fitting the experimental values to the pseudo-first and pseudo-second order models are given in Table 2. The *R*^2^ values for the pseudo-second order kinetic model were higher than those of the pseudo-first order model. In addition, the calculated values of *q_e_* (Table 2) determined by the pseudo-second order model are more consistent with the measured values of *q_e_*. These results prove that the adsorption process of these dyes on Fe_3_O_4_@C@C NPs followed the pseudo-second order kinetic model, suggesting that adsorption is dependent on the amount of the solute adsorbed on the surface of the adsorbent and the number of active sites. Note that if most of the data points are close to equilibrium, as in our case with MB adsorption, linear pseudo-second order modeling will lead to an excellent fit [35]. Indeed, linear modeling of experimental data on the adsorption of MB gives *R*^2^ = 0.99594 and *q_e_* = 10.82954.

The intraparticle diffusion model is often used when considering the liquid/solid adsorption kinetics. Boyd et al. [36] and, a little later, Weber and Morris [37], proposed an intraparticle diffusion equation for the case of spherical particles, according to which the rate of diffusion is the time square root function.

(5) $$q_{t}=k_{i}t^{1/2}+C,\;$$

where *k_i_* is the intraparticle diffusion rate constant, (mg/g.min^1/2^), *C* is the intersection point of the *q_t_* = *t*^1⁄2^ straight line with the *y*-axis. If the adsorption is due to the intraparticle diffusion only, the experimental graph *q_t_*(*t*^1/2^) should be a straight line passing through the origin. Otherwise, if the graph is multilinear or does not pass through the origin, more than one diffusion mechanism determines the adsorption process, and adsorption can also occur by diffusion within the particles.

**Figure 12:**
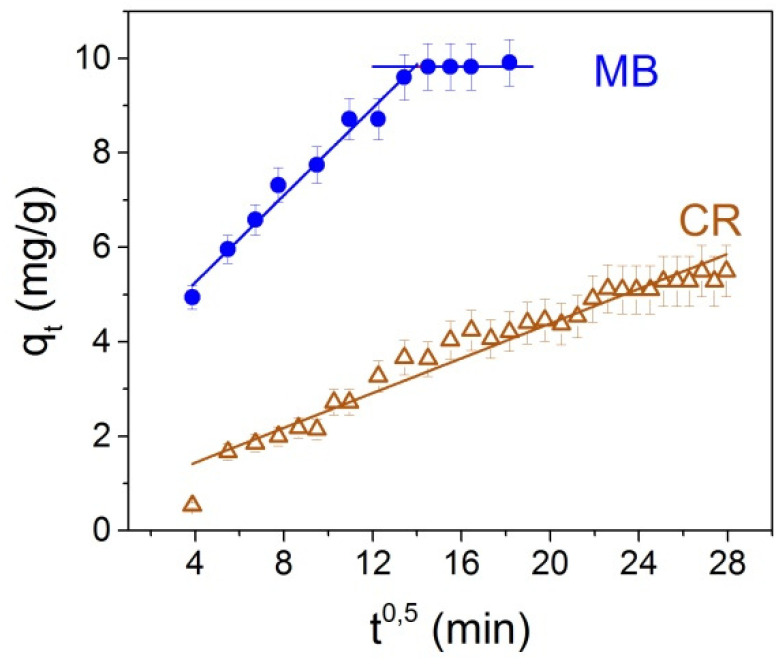
The *q_t_* dependencies *t*^1/2^ calculated with Equation (5) for MB (circles) and CR (triangles).

As can be seen in Figure 12, two linear sections corresponding to two different stages of adsorption are observed in the case of MB adsorption. Therefore, in this case, it is possible to assume two or more diffusion mechanisms responsible for the adsorption. The transport of dye molecules from the solution to the external surfaces of the NPs may be responsible for the first stage, which lasts ~145 min for MB and characterizes the sorption of CR throughout the process. High initial adsorption rates of *k*_1_ of the first stage (Table 2) are observed for MB, indicating a rapid initial process of dye removal and the predominant role of diffusion of the outer surface. The second stage corresponds to the diffusion of dye molecules inside the micropores of Fe_3_O_4_@C@C NPs. Extremely low rates of *k*_2_ adsorption indicate an insignificant proportion of intraparticle diffusion of dye molecules inside NPS micropores. Since in the case of MB, the intersection of the first linear section is far from zero; this suggests that the adsorption of MB on Fe_3_O_4_@C@C NPS includes diffusion between particles and diffusion of the boundary layer. Similar adsorption processes were observed for MB by other authors, for example, [16,22].

To obtain the relationship between the quantity of gas adsorbed into a solid surface and the gas pressure, H. Freundlich proposed in 1909 an empirical expression describing the isothermal variation of a quantity of gas adsorbed by unite mass of solid adsorbent in dependence on gas pressure known now as the Freundlich isotherm [38]. The same equation began to be used to describe the concentration of a solute adsorbed onto the surface of a solid adsorbent.

(6) $$q_{e}=K_{F}C_{e}^{1/n}\;(\mathrm{Freundlich}\;\mathrm{isotherm}),$$

where *q_e_* is the solute amount adsorbed at equilibrium (mg/g), *C_e_* is the concentration of the dye (mg/L), *K_F_* is the constant of adsorption equilibrium, and 1/n is an empirical dimensionless parameter related to the isotherm shape. *K_F_* and n are constants for a given adsorbate and adsorbent at a given temperature.

Langmuir [39] proposed another adsorption model assuming an adsorbate behavior to be similar to ideal gas at isothermal conditions and adsorbent to be similar to an ideal solid surface composed of a series of distinct sites capable of binding the adsorbate. The Langmuir equation (isotherm) is as follows:

(7) $$q_{e}=q_{max}*\frac{K_{L}C_{e}}{K_{L}C_{e}+1}\;(\mathrm{Langmuir}\;\mathrm{isotherm}).$$

Here, *q_max_* is the maximum adsorption capacity; the other parameters coincide with that in Equation (6). Thus, the Langmuir model well describes the monomolecular adsorption and is often applied to a homogeneous adsorbing surface, where all adsorption sites have the same affinity for the adsorbate, while the Freundlich model assumes some heterogeneity of adsorption surfaces.

**Figure 13:**
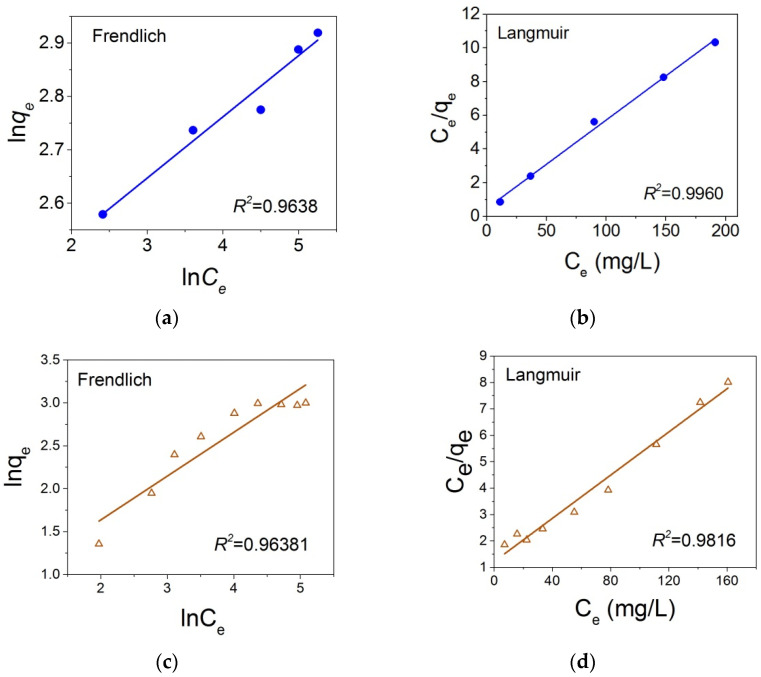
Adsorption Freundlich (**a**,**c**) and Langmuir (**b**,**d**) isotherms in the coordinates of their linear equations for MB (**a**,**b**) and for CR (**c**,**d**) on NPs Fe_3_O_4_@C@C.

According to Equations (6) and (7), *lnq_e_* and *C_e_*/*q_e_* should be linear functions of *lnC_e_* and *C_e_*, correspondingly for the Freundlich and Langmuir models. Corresponding dependencies shown in Figure 13 for the MB and CR adsorption on the Fe_3_O_4_@C@C NPs were obtained at the next experimental conditions: sorbent mass was 3 mg, the dye solution volume was 1.5 mL, contact time was 4 h (for MB) and 8 h (for CR) with periodic exposure to ultrasound for 10 min with intervals of 10 min, temperature controlled by a thermocouple was T = 23–27 °C. As seen from Figure 13, these dependencies are rather close to linear but the experimental points are more consistent with the Langmuir adsorption isotherm than with the Freundlich one. The obtained parameters of the Langmuir isotherms are: *q_e_* = 19.07 (mg/g), *K_L_* = 0.1098, *R^2^* = 0.996 for MB and *q_e_* = 24.44 (mg/g), *K_L_* = 0.0336, *R*^2^ = 0.9816 for CR, the Freundlich isotherms are *K_F_* = 10.0 (mg/g), 1/n = 0.11 (n = 8.71), *R*^2^ = 0.9638 for MB and *K_F_* = 1.84 (mg/g), 1/n = 0.51 (n = 1.96), *R*^2^ = 0.9638. *R*^2^ is the so-called correlation parameter characterizing deviation of experimental points from the straight line. *R*^2^ = 1 corresponds to the full coincident of experimental points and this line. The *R*^2^ values close to 1 and the n value between 1 and 10 in both cases suggests a favorable adsorption potential of the adsorbent.

**Figure 14:**
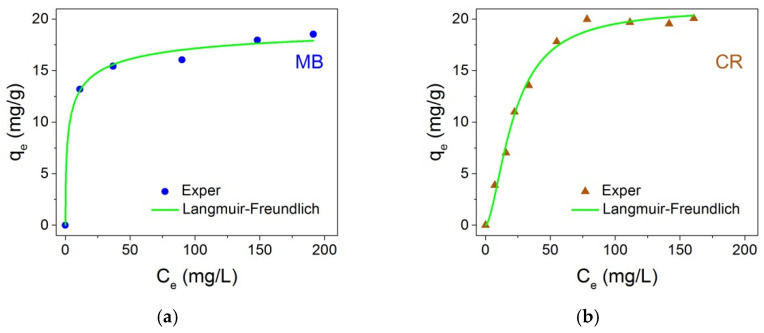
Adsorption isotherms for MB (**a**) and CR (**b**) dyes on the Fe_3_O_4_@C@C NPs at 25 °C.

Recently, a combined adsorption isotherm, which integrates both Langmuir and Freundlich models described by the formulae, has become widespread.

(8) $$q_{e}=\frac{q_{m}{\left(K_{LF}C_{e}\right)}^{n_{LF}}}{1+{\left(K_{LF}C_{e}\right)}^{n_{LF}}},\;$$

where *q_m_* and *K_LF_* denote adsorption capacity and affinity constant, respectively, like similar parameters in the Langmuir model, *n_LF_* is the heterogeneity factor or a measure for the adsorption intensity as in the Freundlich model [40,41,42]. It is accepted that at *n_LF_* < 1 a normal adsorption—Freundlich or Langmuir—takes place, while for *n_LF_* > 1, the Langmuir–Freundlich model (mathematical equivalent of the Hill equation [43] in biochemistry) assumes that the adsorption is a cooperative process influenced by primary and secondary adsorbate–adsorbent, adsorbate–adsorbate interactions. At *n_LF_* = 1, Equation (8) is reduced to Langmuir isotherm indicating the ability of adsorption sites to be not dependent on solution concentration. Experimental adsorption data the MB and CR dyes are shown in Figure 14 together with Langmuir–Freundlich curves. The adsorption parameters, as well as the correlation coefficient of the fitting, are presented in Table 3.

Judging by the value of the *n_LF_* parameter, MB adsorption on NPs occurs mainly according to the Langmuir model, while the combined Langmuir–Freundlich model better describes the adsorption of CR on the same NPs. Possibly, this difference is due to the difference in the MB and CR molecules size: the CR molar mass is 8000 g/mole while the MB molar mass is only 319.85 g/mole. For the smaller MB molecules, the adsorbing NPs surface is homogeneous, predominately, for the much larger CR molecules the same surface looks like heterogeneous. This allows us to suggest that large, regular-shaped particles consist of rather densely packed Fe_3_O_4_@C NPs in the carbon matrix with small pores. This assumption does not contradict the results of the study of the morphology and magnetic properties of Fe_3_O_4_@C@C NPs.

Table 4 summarizes the data on the adsorption capacity of different Fe_3_O_4_-carbon nanostructures for the removal of the anionic and cationic dyes from water obtained here and presented in the literature. The maximum adsorption capacity *q_max_* of the synthesized Fe_3_O_4_@C@C core-shell magnetic nanoparticles is comparable to the data of other authors.

The wide range of values of the adsorption capacity obtained by the adsorption of the same dye, seemingly by the same material—magnetite NPs in carbon, indicates that in each case this process is determined by the structural features of the carbon surface. Almost in all the cases, magnetite—carbon nanostructured materials demonstrate a better adsorbent capacity for cationic dyes.

## 4. Conclusions

In this study, the core-shell magnetic nanoparticles, Fe_3_O_4_@C, were synthesized by the thermal decomposition method in a one-step process. The nanoparticles were used as a starting material to prepare the double carbon coated NPs, Fe_3_O_4_@C@C, by heating them for 12 h in an aqueous glucose solution at a temperature of 200 °C. The X-ray and electron diffraction data showed the magnetic core of the Fe_3_O_4_@C NPs to have the magnetite Fe_3_O_4_ crystal structure without the presence of any other phases. Most NPs in this case were of a spherical shape, and their average crystallite size was estimated to be 43 nm. The amorphous carbon shells were about several nanometers in thickness. As a result of the treatment in the glucose solution, the Fe_3_O_4_@C NPs self-organized into large polyhedral conglomerates 200–300 nm in size of the same morphology, resembling pillows in shape. A number of isolated particles with a magnetic core of the same size as in the first case but with a very thick carbon shell (about 7 nm) were observed along with the large polyhedral conglomerates. XRD analysis and Mössbauer spectroscopy showed Fe_3_O_4_ as the main magnetic phase (approximately 80%) with the admixture of γ-Fe_2_O_3_ (20%). The correct determination of the crystallite size was difficult due to the presence of two phases. However, the comparison of the width of the reflections in the XRD patterns allowed us to assume that the sizes of crystallites in this case were close to that in the NPs coated with carbon once. The results of the XRD, TEM, and magneto-static studies suggested that these polyhedral conglomerates raised around peculiar templates made of interacting glucose molecules under the technological conditions used. The unusually high saturation magnetization Ms revealed by the magnetic measurements should be noted. For Fe_3_O_4_@C NPs it was even higher than the Ms of the bulk magnetite crystal. The high Ms value can be considered an advantage of the studied nanomaterials since a higher magnetization requires the use of weaker magnetic fields to control the processes involving these materials.

The organic dyes adsorption on the obtained NPs has been studied. Two so-called reference dyes analogous to endotoxins—the cationic methylene blue and anionic Congo red—were chosen for the experiment. Seemingly, the Congo red adsorption on the carbon based nanostructures was investigated for the first time.

The kinetics of both dyes’ sorption processes was found to follow the pseudo-second order rate law and equilibrium data agree well with the Langmuir isotherm and therefore the sorption process can be controlled by chemisorption. The studied NPs sorption capacity for CR was shown to be higher compared to that of the carbon based structure capacity for other anionic dye, Cresol Red, studied by several authors. For MB, the maximum adsorption capacity of the Fe_3_O_4_@C@C NPs is comparable to other analogous adsorbents presented in the literature. Thus, the studied NPs can be used as a magnetically detachable and effective adsorbent for environmental protection.

## Figures and Tables

**Table 1:** Mössbauer parameters of the samples Fe_3_O_4_@C and Fe_3_O_4_@C@C at room temperature. IS is a chemical isomeric shift relative to α-Fe, H_hf_ is a hyperfine field on Fe nuclei, QS is quadruple splitting, W is the width of the Mössbauer line at half-height, A is the relative occupancy of the position.

	IS, ±0.005 mm/s	H_hf_, ±5 kOe	QS, ±0.02 mm/s	W, ±0.03 mm/s	A, ±0.03 o.e.	Position/Phase
Fe_3_O_4_@C						
S1	0.668	460	0	0.24	0.64	[Fe]—Fe_3_O_4_
S2	0.283	490	0	0.24	0.36	(Fe)—Fe_3_O_4_
Fe_3_O_4_@C@C						
S1	0.670	456	0.02	0.27	0.50	[Fe]—Fe_3_O_4_
S2	0.254	488	0	0.31	0.22	(Fe)—Fe_3_O_4_
S3	0.356	490	0.0	0.24	0.28	γ-Fe_2_O_3_

**Table 2:** Kinetic parameters for nonlinear modeling adsorption kinetics (Equations (3)–(5)), for the adsorption of MB and CR dyes (initial *C_0_* = 30 mg/L) on Fe_3_O_4_@C@C NPs at 25 °C.

Kinetic Model	Parameters	CR	MB
Pseudo-first order	*k*_1_ (1/min)	0.00638 ± 0.0004	0.0305 ± 0.004
	*q_e_* (mg/g)	5.19 ± 0.10	9.39 ± 0.28
	*R* ^2^	0.9576	0.8366
Pseudo-second order	*k*_2_ (g/(mg min))	0.0011 ± 0.0001	0.004 ± 0.0001
	*q_e_* (mg/g)	6.33 ± 0.13	10.49 ± 0.25
	*R* ^2^	0.9798	0.9482
Intraparticle diffusion	*k_i_*_1_ (mg/(g min^0.5^))	0.1842 ± 0.0077	0.4617 ± 0.012
	*C*_1_ (mg/g)	0.71 ± 0.15	3.41 ± 0.14
	*R* ^2^	0.9521	0.9786
	*k_i_*_2_ (mg/g min^0.5^)		−1.6018 ± 0.001
	*C*_2_ (mg/g)		9.82 ± 0.003
	*R* ^2^		0.9999

**Table 3:** Langmuir–Freundlich adsorption isotherm constants for MB and CR on NPs Fe_3_O_4_@C@C.

Dye	*q_m_* (mg/g)	*K_LF_* (L/mg)	*n_LF_*	*R* ^2^
MB	20.04	3.48	0.53	0.995
CR	21.07	21.82	1.66	0.992

**Table 4:** Comparison of the adsorption capacity of the Fe_3_O_4_@C@C NPs for the anionic and cationic dyes.

Cationic Dye	Adsorbent	*q*_max_ (mg/g)	Anionic Dye	Adsorbent	*q*_max_ (mg/g)
MB	Fe_3_O_4_@C@C ^a^	16 [This work]	CR	Fe_3_O_4_@C@C ^b^	19 [This work]
	Fe_3_O_4_/C core–shell NPs (~250 nm) ^c^	44.4 [34]	Cresol red	Fe_3_O_4_/C core–shell nanoparticles (~ 250 nm)^c^	11.2 [34]
	Fe_3_O_4_@C (~3000 nm) ^d^ B-Fe_3_O_4_@C (~2000 nm) ^e^	15 [10] 40 [10]	Cresol red	Fe_3_O_4_@C (~ 3000 nm) ^d^	11 [10]
	Fe_3_O_4_ NPs (~200 nm)on the graphene layers ^f^	35 [12]			
	Fe_3_O_4_@C submicron rods ^g^	24 [14]			
	Fe_3_O_4_ NPs on glucose-functionalized hydrophilic graphene nanolayers ^h^	18–20 [44]			
	Fe_3_O_4_@C NPs (~30 nm) ^i^	17 [15]			

^a^ *q*_max_ = *q_e_* at C0 = 50 mg/L, adsorbent 0.2 g/L. ^b^ *q*_max_ = *q_e_* at *C_0_* = 70 mg/L, adsorbent 0.2 g/L. ^c^ *C_0_* = 70 mg/L. ^d^ *C_0_* = 20 mg/L, pH = 7.0, adsorbent 0.2 g/L. ^e^ *Co* = 50 mg/L, pH = 7.0, adsorbent 0.2 g/L. ^f^ *Co* = 25 mg/L, adsorbent 0.4 g/L. ^g^ *Co* = 30 mg/L, pH = 7.0, T = 300 K, adsorbent 0.1 g/L. ^h^ *C_0_* = 20 mg/L, T = 300 K, adsorbent 0.1 g/L. ^i^ *C_0_* = 50 mg/L, adsorbent 2.0 g/L.

## Data Availability

The data presented in this study are available on request from the corresponding author. The data are not publicly available since they are a part of ongoing research.
